# CYP3A4*1B but Not CYP3A5*3 as Determinant of Long-Term Tacrolimus Dose Requirements in Spanish Solid Organ Transplant Patients

**DOI:** 10.3390/ijms252011327

**Published:** 2024-10-21

**Authors:** Julia Concha, Estela Sangüesa, María Pilar Ribate, Cristina B. García

**Affiliations:** Department of Pharmacy, Faculty of Health Sciences, Universidad San Jorge, E-50830 Villanueva de Gállego, Zaragoza, Spain; jconcha@usj.es (J.C.); esanguesa@usj.es (E.S.); cbgarcia@usj.es (C.B.G.)

**Keywords:** tacrolimus, transplantation, pharmacogenetics, single nucleotide polymorphism (SNP)

## Abstract

Tacrolimus (TAC) is a commonly used immunosuppressive drug in solid organ transplantation. Pharmacogenetics has been demonstrated before to be decisive in TAC pharmacotherapy. The CYP3A5*3 variant has been reported to be the main determinant of TAC dose requirements; however, other polymorphisms have also proven to be influential, especially in CYP3A5 non-expressor patients. The aim of this study is to evaluate the influence of genetic polymorphisms in TAC therapy in a cohort of Spanish transplant recipients. Genetic analysis including ten polymorphic variants was performed, and demographic and clinical data and pharmacotherapy of 26 patients were analyzed. No significant differences were found in weight-adjusted dose between CYP3A5 expressors and non-expressors (0.047 mg/kg vs. 0.044 mg/kg), while they were found for carriers of the CYP3A4*1B allele (0.101 mg/kg; *p* < 0.05). The results showed that patients with at least one CYP3A4*1B allele had a higher TAC dose and lower blood concentration. Dose-adjusted TAC blood levels were also lower in CYP3A4*1B carriers compared to non-carriers (0.72 ng/mL/mg vs. 2.88 ng/mL/mg). These results support the independence of CYP3A5*3 and CYP3A4*1B variants as determinants of dose requirements despite the linkage disequilibrium present between the two. The variability in genotype frequency between ethnicities may be responsible for the discrepancy found between studies.

## 1. Introduction

Immunosuppressive therapy in solid organ transplantation is necessary to avoid the risk of allograft rejection by the immune system. It depends on the time post-transplant, with the immunosuppressive load being higher in the short term and progressively decreasing until 1 year, when maintenance doses are normally reached. Drug therapy mostly includes a calcineurin inhibitor such as tacrolimus (TAC) or cyclosporine, in addition to an antiproliferative agent such as mycophenolic acid. Corticoids are generally prescribed in the short-term post-transplant but are sometimes maintained during long-term therapy. mTOR inhibitors such as everolimus may be added in order to decrease calcineurin inhibitor dose [1]. A recent systematic review concluded that TAC is superior to cyclosporine in reducing the number of acute rejections, hypertension, and dyslipidemia associated with the use of these drugs. However, it turned out to be inferior in terms of the occurrence of glycemic effects [2]. TAC initial dose requirements mainly depend on the type of transplant and concomitant immunosuppressive therapy, and it must be adjusted in order to obtain TAC expected blood levels and optimal response. During maintenance therapy, the therapeutic range is established between 4 and 8 ng/mL or 3 and 7 ng/mL if mTOR inhibitor coadministration [3]. However, large inter-individual variability remains that may be caused by physiological factors, ethnicity, diseases or concomitant therapies [4]. Genetics has also been shown to be a cause of the variability of response found between individuals. Some genetic variants have been shown to affect TAC dosage or pharmacokinetics or even the risk of adverse drug effects, while there is less scientific evidence regarding pharmacodynamic variables. [5]. Among these, there are polymorphisms of genes that encode for main metabolizing cytochromes, transport enzymes, transcription factors, and other genes that may indirectly affect TAC metabolism.

Specifically, the CYP3A5*3 polymorphism of the *CYP3A5* gene, the main metabolizing cytochrome of TAC, has been shown to be primarily responsible for TAC dose requirements [6]. This is the only polymorphic variant included in the Clinical Pharmacogenetics Implementation Consortium (CPIC) guidelines for TAC at the moment, with an evidence level of 1A in the PharmGKB database; the CPIC guidelines are commonly used to support personalized medicine-implementation projects [7]. The presence of the *3 allele generates a splicing defect and the production of an afunctional protein. Carriers of this mutation in homozygosis do not express this cytochrome (CYP3A5 non-expressors), diverting the metabolism of TAC toward its second major route, the CYP3A4 cytochrome. However, the majority of the European population has a non-CYP3A5 expressing phenotype, with the presence of the *1 allele in only 7.5% of the European population [8]. The CPIC guidelines recommend a TAC 1.5–2-fold increase in the starting dose in CYP3A5 expressors (in homo or heterozygousis), while the Dutch Pharmacogenetics Working Group (DPWG) recommends up to an increase of 2.5 times in the conventional dose. Other non-functional alleles like CYP3A5*6 and CYP3A4*7 are hardly present in the Iberian population examined in the present study [8].

Moreover, polymorphisms of the *CYP3A4* gene have also shown evidence in this regard; however, there is still some controversy, having a level of evidence 2A in PharmGKB in relation to its dosage and pharmacokinetics [9]. The variant with the strongest evidence to date, the *22 allele located in intron 6, is present in 3.7% of the Iberian population and produces a reduction in mRNA production and CYP3A4 cytochrome enzyme activity in the liver, reflecting a need for lower drug dosage and lower risk of overexposure [8,10].

On the other hand, the *1B variant is located in the 5′ promoter region of the *CYP3A4* gene, where transcriptional regulatory elements bind [8]. It seems that the presence of this variant in the gene promoter affects the binding of transcriptional repressors, which are inhibitory proteins of gene transcription that regulate the final expression of the gene [11]. Previously, Amirimani et al. (2003) demonstrated in different cell lines (HepG2 and MCF-7) and primary donor hepatocytes that the presence of the CYP3A4*1B allele was associated with 1.2–1.9 times higher CYP3A enzyme activity [11]. Subsequently, numerous studies have been carried out in patients trying to establish the relationship of this allele with the pharmacokinetics of TAC and its therapeutic response, showing mostly an increased enzyme activity and a higher dose of drug required [12]. A meta-analysis by Shi et al. (2015) showed that the presence of the *1B allele affects the required dose of TAC and weight-adjusted blood levels in kidney transplant patients, specifically in Europe, even independently of *CYP3A5*, although these two tend to be in linkage disequilibrium. [13]. CYP35*1 variant is only present in 7.5% of the Spanish population, and CYP3A4*1B does in just 2.8% [8].

Polymorphisms in other genes have been studied to a greater or lesser extent in their relationship to TAC pharmacokinetics, such as transport proteins or transcription factors. Specifically, p-glycoprotein (p-gp), encoded by the *ABCB1* gene, is an active transport pump responsible for expelling xenobiotics and pharmaceuticals to the cell exterior. This gene has three main variants, c.3435T>C (synonymous variant), c.2677T>G/A (missense variant), and c.1236T>C (synonymous variant), usually known as a haplotype, because of their tendency to be inherited jointly in a high percentage of cases. These three variants have been linked to modifications in the function of p-gp and, in general, a loss of function and substrate specificity of this protein. However, some results remain contradictory even though this protein is known to exert resistance to certain oncological treatments [14]. Pregnane X Receptor gene (PXR) or cytochrome P450 oxidoreductase gene (*POR*) have also been studied for their indirect relationship to TAC metabolism, although they still have little evidence in the scientific literature [15,16].

**Figure 1:**
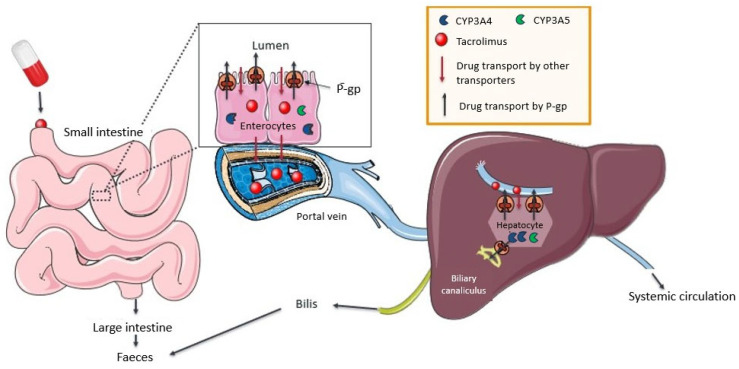
TAC pathways of absorption, metabolism, and excretion.

Absorption, metabolism, and excretion pathways of TAC are depicted in Figure 1.

In addition, transplant patients are often polymedicated patients with comorbidities. Concomitant therapy may also play a role in potential drug-drug interactions. Previously, interactions have been detected with commonly prescribed drugs such as proton pump inhibitors [17]. According to the last Annual Report of the National Health System in Spain in 2022, proton pump inhibitors were the chemical subgroup with the highest consumption, specifically omeprazole. This drug shares a metabolic pathway with TAC, CYP3A4, which becomes predominant in case of alterations in CYP2C19, the main metabolizer enzyme of omeprazole [17].

The aim of this study is to evaluate the influence of genetic polymorphisms of the main metabolizing enzymes, transporter proteins, and transcription factors on TAC pharmacotherapy in a population of Spanish transplant recipients. Furthermore, it is intended to evaluate possible interactions with concomitant therapies that may affect TAC pharmacotherapy.

## 2. Results

### 2.1. Patient Characteristics and Pharmacotherapy

A total of 26 transplant patients were recruited. Patient characteristics are summarized in Table 1.

All patients were white Spanish individuals. There was only 1 case with obesity (BMI > 30), 15 cases (55.5%) with a normal weight (BMI < 25), and no cases that were underweight (BMI < 18.5). Most patients were liver transplants followed by renal transplants.

Regarding patients’ pharmacotherapy, TAC dosage and concomitant drugs are summarized in Table 2.

The range of total daily TAC dose was wide (6.8 mg) with a minimum of 0.2 mg/day and a maximum of 7 mg/day. Regarding the weight-adjusted dose, two values above 0.1 mg/kg were found. No significant differences were found in the daily TAC dose and weight-adjusted daily TAC dose according to age, sex, and TAC release form (*p* = 0.155 and *p* = 0.089, *p* = 0.425 and *p* = 0.951, and *p* = 0.178 and *p* = 0.642 respectively). In relation to the possible induction of CYP3A4 by the concomitant use of corticoids, there was no association between its use (specifically prednisone) and differences in dose (*p* = 0.270) or weight-adjusted dose (*p* = 0.590).

The presence of nephrotoxicity was significantly higher in men than in women (*p* = 0.025). Likewise, there is a non-significant trend in the presence of DM in women compared to men (*p* = 0.082). Weight was related to the presence of HTN, with an average weight of 14.66 kg more in the HTN group than in the non-HTN group, and 3.41 BMI points higher, going from normal weight to overweight. Also, a significant association with HTN and prednisone was found (*p* = 0.008). 8 out of 9 (88.9%) patients with HTN were taking prednisone out of a total of 13 patients with prednisone (61.5%).

### 2.2. Genotyping Results

Observed genotype frequencies are summarized in Table 3.

**Figure 2:**
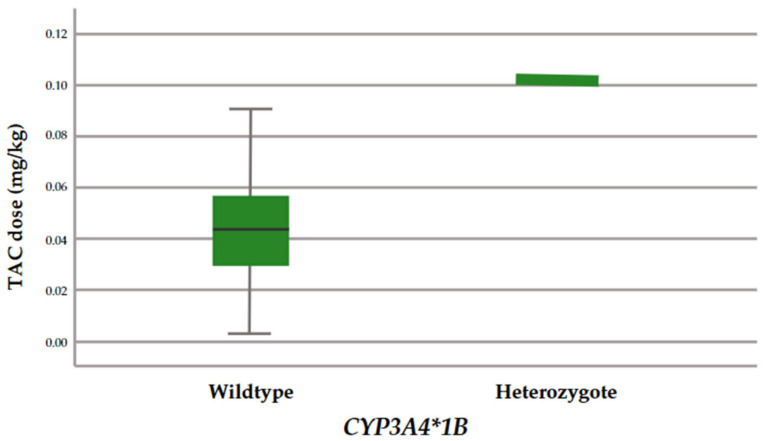
Weight-adjusted dose for each genotypic group (rs2740574).

**Figure 3:**
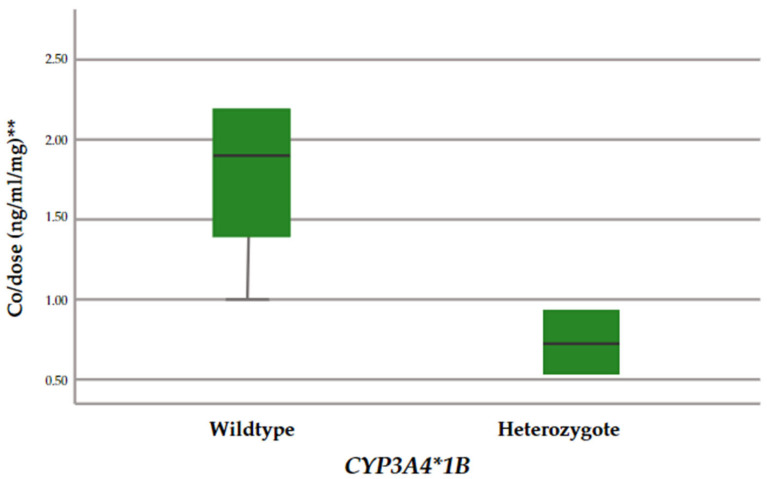
Co/dose for each genotypic group (rs2740574). ** TAC Blood levels were obtained in a limited number of patients.

All the genotypes were in Hardy–Weinberg equilibrium, indicating that there was no population deviation in the study groups (*p* > 0.05). Furthermore, the frequencies of all the genes analyzed were similar to the allele frequencies of the Iberians [8]. Significant differences in weight-adjusted dose (*p* = 0.007) and a trend in Co/dose (*p* = 0.056) were found between CYP3A4*1B allele carriers and non-carriers. Figure 2 and Figure 3 show plots of weight-adjusted dose and Co/dose for the two groups of *CYP3A4* genotypes (rs2740574).

Patients’ phenotypes were classified as fast metabolizers (CYP3A5-expressers and CYP3A4*1B-carriers), intermediate metabolizers (CYP3A5-expressers and CYP3A4*1B-non-carriers), and poor metabolizers (CYP3A5 non-expressors). The data were analyzed according to the combination of the *CYP3A4* and *CYP3A5* genotypes, and differences between groups were obtained (Table 4).

The weight-adjusted dose for poor and intermediate metabolizers was the same for both groups. However, differences were observed with respect to the fast metabolizer group, which had a more than two-fold average dose. The same differences were observed for the total daily dose. When Co/dose were compared, CYP3A4*1B allele carriers had four-fold fewer values compared to non-carriers.

Regarding adverse effects, significant associations were found between CYP3A4*1B and *PXR* 69789GG variants and impaired liver function (*p* = 0.040 and *p* = 0.049, respectively). Considering both variants, 66.66% of the cases were carriers of the CYP3A4*1/*1B-PXR 69789GG combination, all of whom were liver transplant recipients.

## 3. Discussion

The use of prednisone as a concomitant treatment and being overweight were associated with the presence of hypertension in our cohort. It is assumed that the hypertensive potential of TAC is enhanced by the presence of long-term corticosteroid treatment, increasing the likelihood of hypertension in long-term transplant recipients. Likewise, being overweight is a well established cause of high blood pressure involving several pathways, including stimulation of the renin–angiotensin–aldosterone system [18].

Pharmacogenetics has robustly demonstrated that it can affect the pharmacokinetics of TAC. Currently, there is sufficient data that show that the use of *CYP3A* genotyping to establish the initial dose of TAC may be beneficial in terms of reducing the number of dose modifications and time to achieve target blood levels.

However, there is still not enough clinical evidence to determine that its implementation at a clinical level represents clinical improvements over TAC pharmacokinetic monitoring. That is why, currently, in Spain, it is not included in the service portfolio of most hospitals.

Although many genetic polymorphisms present in the five selected genes related to TAC pharmacokinetics have been analyzed, no significant differences have been found in these variants except for *CYP3A4*, despite other previous studies that have shown their relationship [19,20,21,22,23,24,25].

The main polymorphic variants studied are involved in cytochrome activity and expression, most notably the *CYP3A5* gene. Recently, a GWAS study in 251 Chinese kidney transplant recipients evaluating more than 773,000 SNPs concluded that CYP3A5*3 genotype accounted for an intervariability in TAC levels of >37% [25]. The CYP3A4*1B variant has been associated with higher enzyme activity and, therefore, a fast metabolizer phenotype, requiring higher doses in these patients to reach the target therapeutic range, especially in the long post-transplant period [26]. Although its association is not as well established as in the case of CYP3A5*3, there are numerous studies on this variant showing its influence on TAC pharmacotherapy, even if some fail to demonstrate an association [9]. It has been shown that there is a strong linkage disequilibrium between these two SNPs [27]. Thus, some studies attribute the effect of the CYP3A4*1B allele to an increased metabolism generated by the co-presence of CYP3A5*1 [28,29]. However, several authors have determined that the influence of CYP3A4*1B on TAC pharmacokinetics is independent of the *CYP3A5* genotype [30].

It is possible that the genotypic frequency observed in different groups is responsible for the variations found between studies. In a review by Tavira et al. (2014) evaluating kidney transplant recipients from Spain, Japan, and France, significant differences in weight-adjusted dose according to *CYP3A5* genotype were observed. Specifically, CYP3A5 expressors in homozygosis had up to twice the weight-adjusted dose than non-expressors, and CYP3A5 expressors in heterozygosis had up to 50% more weight-adjusted dose. Moreover, there were also large differences in weight-adjusted doses for the same *CYP3A5* genotype among these three populations, being higher in the French than in the Japanese and in both compared to the Spanish one [31]. These results were corroborated by a subsequent meta-analysis including a total of 1182 adult renal patients demonstrating the influence of CYP34*1B on TAC weight-adjusted dose and Co/dose, especially in the European population [13]. In our study, no significant differences were found in weight-adjusted dose between CYP3A5 expressors and non-expressors; however, they were found for the two carriers of the CYP3A4*1B allele, who were also CYP3A5 expressors (fast metabolizers) in heterozygosity due to the linkage disequilibrium present between these two variants [27]. While based on previous literature, a lower weight-adjusted dose was expected for fast and intermediate versus poor metabolizers, no differences were observed in our study between the latter two. Although some studies have shown the independence of CYP3A4*1B polymorphism from CYP3A5*3 in TAC dose determination, usually *CYP3A5* showed as well to be an independent determinant of dose requirements. In our study, no differences were found between CYP3A5 expressors and non-expressors when studied independently of CYP3A4*1B. Specifically, there were only two patients with weight-adjusted doses higher than 0.10 mg/kg, who were the CYP3A4*1B carriers. These patients also showed the only Co/dose values below 1 ng/mL/mg of the cohort, being likewise outside the therapeutic range (<4 ng/mL/mg). Both patients presented signs of rejection or graft dysfunction. In particular, a recent review linked Co/dose <1 ng/mL/mg with increased incidence of adverse effects and infections, especially associated with fast metabolizing patients [32].

However, there are multiple publications that discuss the challenges of clinical implementation of PGx in liver transplant recipients, as the genotype of the transplanted liver may differ from that of the recipient [33]. Such an analysis was beyond the scope of this study since a sample of the transplanted organ would be necessary.

In relation to adverse effects, CYP3A4*1B and *PXR* 69789GG have been associated in our cohort with impaired liver function. Other authors have associated the *PXR* GG genotype with drug-induced liver injury and elevated transaminase values [34,35].

In previous years, PXR has been explored as an important target in drug-induced liver injury and liver disease. Although the mechanisms responsible for PXR-mediated liver injury need further investigation, some studies have shown that PXR activation can increase the expression of PXR target genes, including those encoding liver enzymes, transporters, and other enzymes involved in biosynthetic pathways, leading to the accumulation of toxic metabolites or endogenous intermediates in the liver. It should be noted that PXR not only regulates drug metabolism but also a wide variety of endogenous pathways [36,37]. Hence, the activation of hepatic metabolism produced by these two polymorphisms seems to be responsible for the impaired liver function; however, these results were not found in the case of CYP3A5-expressers CYP3A4*1B-non-carriers.

In relation to omeprazole and other proton pump inhibitors, no association was found in our cohort between possible drug-drug interactions in patients taking omeprazole and CYP2C19 alterations, as previous studies have shown [17].

Therefore, in CYP3A4*1B carriers, a dose adjustment or an alternative therapy may be necessary, especially when certain clinical factors are present. However, alternative drugs like cyclosporine, sirolimus, or everolimus are metabolized and influenced by the same pharmacogenetic pathways [38].

The present study has certain limitations. First, our sample size was relatively small because the sample source depended on the interests of the participants coming from an association of liver transplant patients (AETHA) and pharmacy office. Furthermore, the collection of TAC blood levels could only be obtained from a limited number of patients. Moreover, this is an observational study, and the absence of a clinical trial design could be another limitation. It would also be interesting to have the genetic profile of the donor as it could be a determining factor to take into account.

## 4. Materials and Methods

### 4.1. Patient Enrolment and Data Collection

Organ solid transplant patients recruited for the study were from the association of liver transplant patients of Aragón (AETHA) and a project carried out in pharmacy offices in Spain. The inclusion criteria included renal, hepatic, cardiac, or pulmonary graft recipients, and recipients of oral TAC treatment. The exclusion criteria included having any other autoimmune disease or diseases for which treatment with immunosuppressive drugs was required. Demographic, clinical, and pharmacotherapeutic data and the prevalence of frequent adverse effects of TAC were collected, including nephrotoxicity, neoplasms, hyperglycemia, diabetes mellitus (DM), dyslipidemia, hypertension (HTN), impaired liver function, gastrointestinal effects, neurotoxicity, and recurrent infections. The “required dose” reflected was the most usual stable dose during the patient’s current maintenance therapy (>1 year), and TAC trough blood levels (Co) were those found at this dose. TAC (Co) were analyzed using chemiluminescent immunoassay (MEIA, Abbott Diagnostics, Chicago, IL, USA). The study was approved by the Regional Ethics Committee (CEICA). All participants signed the informed consent form.

### 4.2. Genotype Analysis

DNA was obtained through a minimally invasive procedure using a fingertip and deposition of a drop of blood on a Whatman FTA™ card (Sigma-Aldrich, St. Louis, MO, USA) DNA extraction was conducted using 5% Chelex^®^-100 resin (Sigma-Aldrich, St. Louis, MO, USA) [39]. A total of ten polymorphic variants present in five genes were analyzed including main metabolizing enzymes and transporter protein variants of TAC: c.6986A>G (rs776746) of the *CYP3A5* gene (NM_000777.5); g.-290A>G (rs2740574), c.15389C>T (rs35599367) and g.87925_87926insA (rs67666821, p.Pro488Thr*fs494) of the *CYP3A4* gene (NM_001202855.3); c.3435T>C (rs1045642, p.Ile1145=), c.2677T>G/A (rs2032582, p.Ala893Thr/Ser) and c.1236T>C (rs1128503, p.Gly412=) of the *ABCB1* gene (NM_000927.3); c.63396C>T (rs2472677) and c.69789A>G (rs7643645) of Pregnane X Receptor (*PXR*) (NM_022002.2); and c.1508C>T (rs1057868, p.Ala503Val) of the cytochrome P450 oxidoreductase (*POR*) gene (NM_000941.3). *CYP2C19* gene (NM_000769.4) main variants, c.681 G>A (rs2472677, c.636 G>A (rs4986893), and c.-80C>T (rs12248560), were studied in relation to the pharmacokinetics of concomitant treatments (i.e., omeprazole). Genotyping was performed using Polymerase Chain Reaction Restriction Fragment Length Polymorphism (PCR-RFLP; MJ MiniTM Personal Thermal Cycler Bio-Rad^®^ Laboratories, Hercules, CA, USA), sequencing (Macrogen, Seoul, Republic of Korea), specific TaqMan™ (Applied Biosystems, Foster City, CA, USA), or rhAmp^®^ (IDT, Newark, NJ, USA) genotyping systems on CFX Connect™Systems (Bio-Rad Laboratories, Hercules, CA, USA) depending on the SNP. Assay references and fluorophores obtained for each allele for TaqMan™ (Thermo Fisher Scientific, Waltham, MA, USA) and rhAmp^®^ (Integrated DNA Technologies, Coralville, IA, USA) methods are detailed in Table 5.

The insertion in position 87,925 (rs67666821) of the *CYP3A4* gene was analyzed with a sequencing method using the primers forward 5′-GAAGGAGTGTCTCACTCA-3′ and reverse 5′-GAGGTCTCTGGTGTTCTCAG-3′ under the following thermocycling conditions: initial denaturalization of 94 °C 10′, followed by (94 °C 30″, 52 °C 30″, 72 °C 90″) × 35 cycles, and a final extension of 72 °C 5′ [40]. c.63396C>T polymorphism of PXR gene was analyzed with PCR-RFLP, using forward primer 5′-TGCTAGCAGTGCATAAGGGCTCAG-3′ and reverse primer 5′-TCCTGACCTTAGGTGATCCATGCC-3′ and the restriction endonuclease *Hpy188I* (37 °C overnight), under this specific thermocycling conditions: initial denaturalization of 94 °C 3′, followed by (94 °C 15″, 60 °C 15″, 72 °C 30″) × 35 cycles, and a final extension of 72 °C 5′ [41]. c.636 G>A variant of CYP2C19 gene was analyzed with PCR-RFLP, using primer forward 5′-AAATTGTTTCCAATCATTTAGCT-3′ and primer reverse 5′-ACTTCAGGGCTTGGTCAATA-3′ and the restriction endonuclease *BamHI* (37 °C overnight), under this specific thermocycling conditions: initial denaturalization of 94 °C 10′, followed by (94 °C 20″, 58 °C 30″, 72 °C 20″) × 36 cycles, and a final extension of 72 °C 6′ [42].

### 4.3. Statistical Analysis

Statistical analysis was performed using SPSS^®^ software version 28.0.1.0 (IBM^®^, Armonk, NY, USA). Non-parametric tests were performed to analyze data. Statistical significance is accepted for a 95% confidence interval (*p* ≤ 0.05). The Hardy–Weinberg equilibrium test was done, and genotypic frequencies were compared with other cohorts of the Iberian population [8].

## 5. Conclusions

The pharmacogenetics of immunosuppressive therapy has been extensively studied. In the case of TAC, genetic variants related to pharmacokinetics have the strongest scientific evidence. Specifically, polymorphisms of the major metabolizing cytochromes have been shown to be the main influencers on TAC pharmacotherapy. Although results may vary between populations due to the diversity of allele frequencies, the CYP3A5*3 variant has been the most widely described as a determinant of TAC dose requirements. CYP3A4*1B has also independently demonstrated its influence in previous literature. However, both variants tend to be co-inherited in a high percentage due to linkage disequilibrium between them. For this reason, some authors attribute the effect of CYP3A4*1B to its linkage with CYP3A5*1, while others justify its independence. In our study, the *1B variant in the *CYP3A4* gene seems to be the major determinant of weight-adjusted dose, whereas CYP3A5*1 showed no influence. Specifically, only the two patients who were carriers of the *1B variant who were also CYP3A5-expressors showed higher weight-adjusted doses than CYP3A5 expressors not carrying the *1B variant. Likewise, the presence of the CYP3A4*1B and *PXR* 69789GG genotypes have been associated with decreased liver function, probably subsequent to the increased hepatic metabolism associated with these variants, as previous studies have also found.

It can, therefore, be concluded that in our cohort, the two patients with at least one CYP3A4*1B allele had a higher TAC dose and lower blood concentration. However, further studies with a higher number of patients are still needed to clarify the association or independence of these two variants due to the linkage disequilibrium found between them.

## Figures and Tables

**Table 1:** Demographic data of the study population.

Demographic Characteristic		Value
Age (Years)		51.04 (±19.26); 52 (27)
Sex	Male	10 (38.5)
	Female	16 (61.5)
Weight (kg)		64.93 (±14.0); 66 (25.3)
BMI (kg/m^2^)		23.89 (±3.8); 24.09 (7.0)
Transplant Type	Hepatic	14 (53.8)
	Renal	9 (34.6)
	Cardiac	2 (7.7)
	Pulmonary	1 (3.8)
Signs of Allograft Rejection	Acute	5 (19.2)
	Chronic	5 (19.2)
	General	9 (34.6)
Re-transplantation		2 (7.4)

Categorical variables are expressed as n (%), and continuous variables are expressed as the mean (standard deviation) and median (interquartile range [IQR]).

**Table 2:** Variables related to TAC treatment.

Variable		Value
Total Daily Dose (mg)		3.04 (±1.55); 3 (2.0)
Weight-Adjusted Dose (mg/kg)		0.049 (±0.025); 0.047 (0.026)
Co (ng/mL) *		4.77 (±1.98); 4.20 (2.7)
Co/Dose (ng/mL/mg) *		2.48 (±3.21); 1.40 (1.2)
Co/Weight-Adjusted Dose (ng/mL/mg/kg) *		175.87 (±275.6); 83 (80.3)
Concomitant Therapy	Mycophenolic acid (MFA)	13 (50)
	Corticoids	13 (50)
	Proton pump inhibitors (excluded rabeprazole)	12 (44.4)
	Antihypertensives	10 (38.5)
	Ursodeoxycholic acid	4 (14.8)
	Statins	4 (14.8)
	mTOR inhibitors	1 (3.7)

Categorical variables are expressed as n(%), and continuous variables are expressed as the mean (standard deviation) and median (interquartile range [IQR]). * TAC blood levels were obtained in a limited number of patients (9 out of 26).

**Table 3:** Observed genotype frequencies of patients of the study.

SNP (rs Number)	Genotype	Observed Frequency, N (%)
CYP3A5*3 (rs776746)	AG	4 (16)
	GG	22 (84)
CYP3A4*1B (rs2740574)	AA	24 (92)
	AG	2 (8)
CYP3A4*22 (rs35599367)	CC	24 (92)
	CT	2 (8)
CYP3A4*20 (Ins A) (rs67666821)	No Ins	26 (100)
POR*28 *(rs1057868)*	CC	12 (46)
	CT	12 (46)
	TT	2 (8)
*ABCB1 (3435C>T)* *(rs1045642)*	CC	8 (30)
	CT	13 (50)
	TT	5 (20)
*ABCB1 (1236C>T)* *(rs1128503)*	CC	6 (23)
	CT	16 (61)
	TT	4 (16)
*ABCB1 (2677G>T/A)* *(rs2032582)*	GG	8 (30)
	GT/GA	15 (57)
	TT	3 (13)
*PXR (c.69789A>G)* *(rs7643645)*	AA	8 (30)
	AG	12 (46)
	GG	6 (24)
*PXR (c.63396C>T)* *(rs2472677)*	CC	6 (23)
	CT	8 (30)
	TT	12 (47)
CYP2C19*2 *(rs4244285)*	GG	18 (69)
	GA	7 (27)
	AA	1 (4)
CYP2C19*3 *(rs4986893)*	GG	26 (1)
	GA	0 (0)
	AA	0 (0)
CYP2C19*17 *(rs12248560)*	CC	19 (73)
	CT	6 (23)
	TT	1 (4)

**Table 4:** Dose and weight-adjusted dose in CYP3A4 and CYP3A5 genotype combination.

Genotype (n)		Dose (mg)	Weight-Adjusted Dose (mg/kg)	Co/Dose (ng/mL/mg) **
CYP3A5*3/*3	CYP3A4*1/*1 (22)	2.72 (1.35)	0.044 (0.021)	2.88 (3.02)
CYP3A5*3/*1	CYP3A4*1/*1 (2)	3.5 (0.70)	0.047 (0.04)	-
	CYP3A4*1/*1B (2)	5.5 (2.12)	0.101 (0.001)	0.72 (0.28)

Data is expressed as mean (SD). ** Co were obtained in a limited number of patients (9 out of 26).

**Table 5:** Methodology and assay references for the polymorphisms analyzed in this study.

Gen	Variant	Methodology/ID Assay	Fluorophore or Primers (Endonuclease)
*CYP3A5* (NM_000777.5)	rs776746	rhAmp^®^/Hs.ADME.rs776746.C.1	FAM: A (*1) VIC: G (*3)
*CYP3A4* (NM_001202855.3)	rs2740574	rhAmp^®^/Hs.CT.rs2740574	FAM: G (*1B) VIC: A (*1)
*CYP3A4* (NM_001202855.3)	rs35599367	TaqMan™/C_59013445_10	FAM: *22 VIC: *1
*POR* (NM_000941.3)	rs1057868	rhAmp^®^/Hs.GT.rs1057868.T.1	FAM: C VIC: T
*ABCB1* (NM_000927.3)	rs1045642	rhAmp^®^/Hs.ADME.rs1045642.T.1	FAM: T VIC: C
*ABCB1* (NM_000927.3)	rs1128503	rhAmp^®^/Hs.ADME.rs1128503.G.11	FAM: T VIC: C
*ABCB1* (NM_000927.3)	rs2032582	TaqMan™/C_11711720D_40	FAM: T VIC: G
*PXR* (NM_022002.2)	rs7643645	rhAmp^®^/Hs.GT.rs7643645.G.a1	FAM: A VIC: G
CYP2C19*2 *(NM_000769.4)*	rs4244285	rhAmp^®^/Hs.ADME.rs4244285.A.1	FAM: G VIC: A
CYP2C19*17 *(NM_000769.4)*	rs12248560	TaqMan™/C_469857_10	FAM: *17 VIC: *1

## Data Availability

The original contributions presented in the study are included in the article. Further inquiries can be directed to the corresponding author.
